# Improving ^18^F-FDG PET Quantification Through a Spatial Normalization Method

**DOI:** 10.2967/jnumed.123.267360

**Published:** 2024-10

**Authors:** Daewoon Kim, Seung Kwan Kang, Seong A. Shin, Hongyoon Choi, Jae Sung Lee

**Affiliations:** 1 Interdisciplinary Program of Bioengineering, Seoul National University, Seoul, South Korea;; 2 Artificial Intelligence Institute, Seoul National University, Seoul, South Korea;; 3 Brightonix Imaging Inc., Seoul, South Korea;; 4 Institute of Radiation Medicine, Medical Research Center, Seoul National University College of Medicine, Seoul, South Korea; and; 5 Department of Nuclear Medicine, Seoul National University College of Medicine and Seoul National University Hospital, Seoul, South Korea

**Keywords:** brain PET, quantification, spatial normalization, glucose metabolism

## Abstract

Quantification of ^18^F-FDG PET images is useful for accurate diagnosis and evaluation of various brain diseases, including brain tumors, epilepsy, dementia, and Parkinson disease. However, accurate quantification of ^18^F-FDG PET images requires matched 3-dimensional T_1_ MRI scans of the same individuals to provide detailed information on brain anatomy. In this paper, we propose a transfer learning approach to adapt a pretrained deep neural network model from amyloid PET to spatially normalize ^18^F-FDG PET images without the need for 3-dimensional MRI. **Methods:** The proposed method is based on a deep learning model for automatic spatial normalization of ^18^F-FDG brain PET images, which was developed by fine-tuning a pretrained model for amyloid PET using only 103 ^18^F-FDG PET and MR images. After training, the algorithm was tested on 65 internal and 78 external test sets. All T_1_ MR images with a 1-mm isotropic voxel size were processed with FreeSurfer software to provide cortical segmentation maps used to extract a ground-truth regional SUV ratio using cerebellar gray matter as a reference region. These values were compared with those from spatial normalization-based quantification methods using the proposed method and statistical parametric mapping software. **Results:** The proposed method showed superior spatial normalization compared with statistical parametric mapping, as evidenced by increased normalized mutual information and better size and shape matching in PET images. Quantitative evaluation revealed a consistently higher SUV ratio correlation and intraclass correlation coefficients for the proposed method across various brain regions in both internal and external datasets. The remarkably good correlation and intraclass correlation coefficient values of the proposed method for the external dataset are noteworthy, considering the dataset’s different ethnic distribution and the use of different PET scanners and image reconstruction algorithms. **Conclusion:** This study successfully applied transfer learning to a deep neural network for ^18^F-FDG PET spatial normalization, demonstrating its resource efficiency and improved performance. This highlights the efficacy of transfer learning, which requires a smaller number of datasets than does the original network training, thus increasing the potential for broader use of deep learning–based brain PET spatial normalization techniques for various clinical and research radiotracers.

## Visual Abstract

**Figure:**
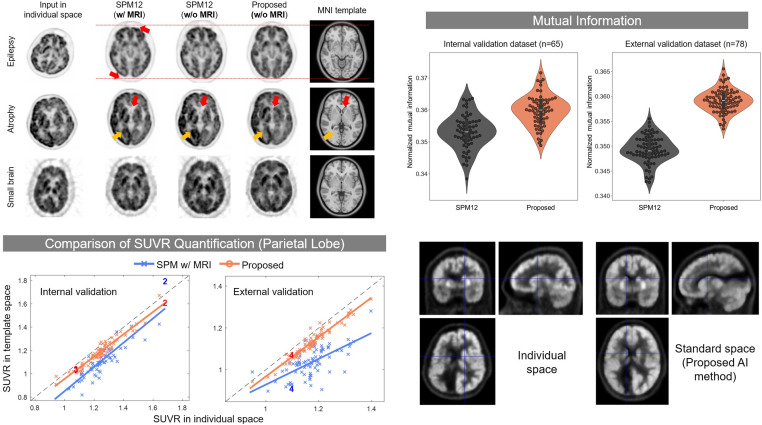

PET is a widely used medical imaging technique for the clinical assessment of patients, focusing on functional and metabolic changes associated with various diseases. Among the radiotracers available for clinical use, FDG labeled with ^18^F (^18^F-FDG) stands out as the most widely used for numerous applications (1–6).

Accurate quantification of ^18^F-FDG PET images is essential for the precise evaluation of various brain diseases, including brain tumors, epilepsy, dementia, and Parkinson disease (7–10). Matched 3-dimensional (3D) T_1_ MRI scans of the same individuals are sometimes used to provide detailed information on brain anatomy, enabling accurate quantification of brain PET images. Alternatively, PET spatial normalization, which does not require matching 3D MR images, has also been widely adopted for brain PET quantification (11–14). However, the accuracy of conventional PET-only spatial normalization, particularly for brain PET images from different ages and ethnic groups than the standard template, is limited, especially in the presence of abnormal ^18^F-FDG uptake due to various pathologic conditions.

Evolutionary advances in artificial intelligence technologies in recent years have had a significant impact on medical image processing and analysis (15). Deep learning–based approaches have emerged that reduce statistical noise, improve the image quality of PET (16–18), and enable rapid quantification of PET and SPECT images without labor-intensive manual drawing of regions of interest (19–22). In addition, artificial intelligence–based methods have been actively explored for PET and SPECT attenuation and scattering correction, radiation dose map generation, and PET detector performance improvement (23–30).

Accurate brain PET quantification without the need for 3D MRI presents another area in which artificial intelligence–based approaches hold great potential. A novel MRI-free brain PET spatial normalization method based on cascaded deep neural networks is proposed (19), demonstrating a superior performance compared with that of conventional MRI-based amyloid PET spatial normalization. However, the requirement for a large number of PET and MR image pairs for deep neural network training presents potential limitations to extending the method to various other brain PET radiotracers.

Our primary objective in this study was to explore the feasibility of transferring a pretrained deep neural network model (31), specifically trained on a large dataset of amyloid PET and paired MR images, for spatial normalization of ^18^F-FDG PET images. Specifically, the pretrained network parameters were fine-tuned using a substantially smaller dataset consisting of around 100 paired ^18^F-FDG PET and MR images. To assess the performance of the transfer learning approach, we used an external dataset from a different ethnic group than the fine-tuning dataset, as well as an internal dataset acquired within the same institution where the fine-tuning dataset originated.

This investigation is important because it can pave the way for more efficient and accurate quantification of brain PET images without the requirement for extensive paired PET and MR image datasets. The successful application of transfer learning could promote the widespread adoption of artificial intelligence–based methods in brain PET imaging, ultimately benefiting patients and advancing medical research.

## MATERIALS AND METHODS

### Datasets

The dataset used for training the original deep neural network model consisted of paired multicenter amyloid PET scans (^18^F-florbetaben or ^18^F-flutemetamol) and structural T_1_-weighted 3D MRI scans from 994 individuals diagnosed with Alzheimer disease or mild cognitive impairment, as well as subjects who were cognitively normal (19). The image data were collected from 6 university hospitals located in South Korea.

For transfer learning and internal evaluation of the network model, we used 168 ^18^F-FDG PET scans and their corresponding paired 3D T_1_ MR images acquired in the Seoul National University Hospital, South Korea. Among the 168 image pairs, 103 were used for fine-tuning the network parameters, whereas the remaining 65 were reserved for performance evaluation. The PET/CT scans were conducted 30 min after the injection of 5.18 MBq/kg of ^18^F-FDG. Emission PET scans were acquired for 8 min using a time-of-flight PET/CT scanner (Biograph 40 or mCT; Siemens Healthineers), followed by CT scans for attenuation correction. PET images were reconstructed using the ordered-subset expectation maximization algorithm with 21 subsets and 5 iterations with a matrix size of 336 × 336 × 148 with voxel sizes of 1.02 × 1.02 × 1.50 mm, and a gaussian postreconstruction filter was applied, with a kernel size of 4 mm. The typical dimension of the T_1_ MR images was 208 × 256 × 256, with voxel sizes of 1.00 × 0.98 × 0.98 mm. The retrospective use of the scan data and waiver of consent were approved by the institutional review board of Seoul National University Hospital.

For additional validation of the proposed method, an external dataset was used, consisting of 78 ^18^F-FDG PET scans, along with their matched T_1_ MRI scans, which are publicly available from OASIS datasets (32). Dynamic ^18^F-FDG PET scans were performed for 60 min using an HR+ PET scanner (Siemens Healthineers) after approximately 5 intravenous injections of 185 MBq of ^18^F-FDG. Emission data were reconstructed into a 128 × 128 × 63 matrix (2.12 × 2.12 × 2.43 mm) using a filter backprojection algorithm. For PET analysis, static PET images generated by integrating dynamic PET frames between 40 and 60 min were used. The matrix size of T_1_ MR images was 256 × 256 × 256 with a 1.00-mm isotropic voxel size.

### Transfer Learning and Evaluation

As previously mentioned, we used a pretrained deep neural network model for spatial normalization, using 994 multicenter and multitracer amyloid PET images, and fine-tuning was conducted using an additional set of 103 internal ^18^F-FDG PET images. Consequently, the network model retains the same structure and parameter numbers as the original model designed for amyloid PET spatial normalization (19). The network architecture comprises cascaded U-Nets, which efficiently generate deformation fields (output) necessary for the nonlinear spatial normalization of input brain PET images resampled to have the dimension of 208 × 256 × 256 and the voxel size of 2 × 2 × 2 mm. The AdamW optimizer (PyTorch) was used to train the network, and the batch size and initial learning rate were 4 and 10^−5^, respectively. The number of epochs was 50, and the learning rate decreased by one tenth every 50 epochs.

During the training stage, these deformation fields were applied to the corresponding paired MR images, allowing us to evaluate the spatial normalization performance by comparing the spatially normalized MR images with the MRI standard template. Subsequently, during the inference stage, only the PET image input was required, enabling rapid MRI-less brain PET spatial normalization with the deformation fields generated by the deep neural networks.

To address potential network parameter overfitting due to the limited number of fine-tuning datasets, we implemented on-the-fly data augmentation techniques. In each iteration of network parameter tuning, input PET images were reshaped and rescaled through random affine transformations, nonlinear deformations, and intensity scaling. This augmentation approach significantly enhanced the generalization capability of the trained network.

After network training, we rigorously evaluated the performance of the spatial normalization network on both internal and external test sets. As mentioned earlier, the internal test set consisted of 65 ^18^F-FDG PET images along with matched T_1_ MR images obtained from our institution. Additionally, we used an external test set comprising 78 ^18^F-FDG PET images and matched T_1_ MR images from the OASIS datasets. The demographics of internal and external validation datasets are summarized in Table 1.

**TABLE 1.** Patient Demographics for Internal and External Validation Datasets

Dataset	*n*	Age (y)	M:F
Internal	65	47.15 ± 21.5	32:33
External	78	61.31 ± 7.39	26:52

To quantitatively evaluate the spatial normalization performance, we measured normalized mutual information between the MNI standard template of T_1_ MRI and the spatially normalized PET images by the proposed method. The spatial normalization performance of proposed method was compared with SPM software (Statistical Parametric Mapping, version 12; https://www.fil.ion.ucl.ac.uk/spm) (33,34). For spatial normalization using SPM, the estimated deformation fields were used to resample PET images in the template space. The deformation fields were estimated in 2 different ways, with or without the assistance of MRI. For the deformation field estimation with MRI, PET and MR images were coregistered initially. Subsequently, MR images underwent spatial normalization to be matched with the standard T_1_ MRI template. Finally, the estimated spatial normalization parameters derived from MR images were applied to the PET image. No preprocessing, such as skull stripping, on the individual MRI has been performed before the MRI spatial normalization, to the best of our knowledge. For the deformation field estimation without MRI, input PET images were nonlinearly registered to a standard PET template without the assistance of MR images.

### ^18^F-FDG Uptake Quantification

In our current study, we followed a methodology similar to that in our previous work with amyloid PET. For validation of the proposed method, PET quantification was conducted in 4 different ways: in individual space with volumes of interest delineated on coregistered paired MRI (ground truth), in standard template space using SPM spatial normalization with MRI, in standard template space using SPM spatial normalization without MRI, and in standard template space using proposed deep learning–based spatial normalization without MRI.

For all T_1_ MR images, we used FreeSurfer 7.1.0 software (Martinos Center for Biomedical Imaging) to obtain cortical segmentation maps using the recon-all function (35). These maps were used for extracting the ground-truth regional SUV ratio (SUVR) with cerebellar gray matter serving as the reference region. These MRI-based SUVRs quantified in individual space were to compare SUVRs with quantification methods based on spatial normalization, including SPM and our proposed method. The T_1_ MRI template was segmented using FreeSurfer to extract SUVRs in the template space.

## RESULTS

**FIGURE 1.**
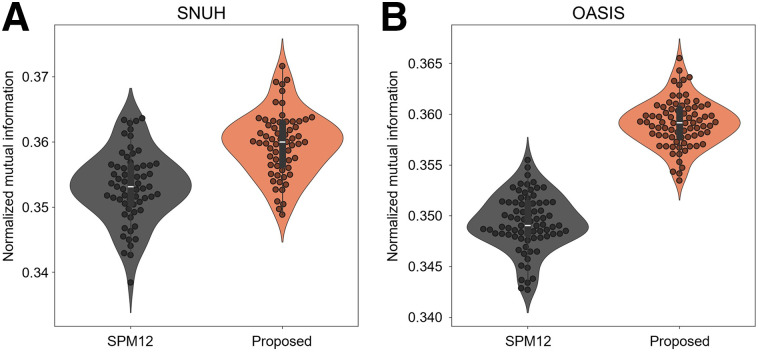
Agreement between spatially normalized images with standard template in terms of normalized mutual information: internal validation dataset (*n* = 65) (A) and external validation dataset (*n* = 78) (B). SNUH = Seoul National University Hospital; SPM12 = SPM version 12.

The proposed deep learning–based method successfully generated spatially normalized PET images without the need for 3D MRI input and provided quantified regional SUVRs. Moreover, the proposed method outperformed SPM spatial normalization, which used 3D T_1_ MRI, for both test datasets. Figure 1 shows that the proposed method achieves remarkably higher agreement with the standard template in terms of normalized mutual information than does the SPM approach on both internal and external validation datasets.

**FIGURE 2.**
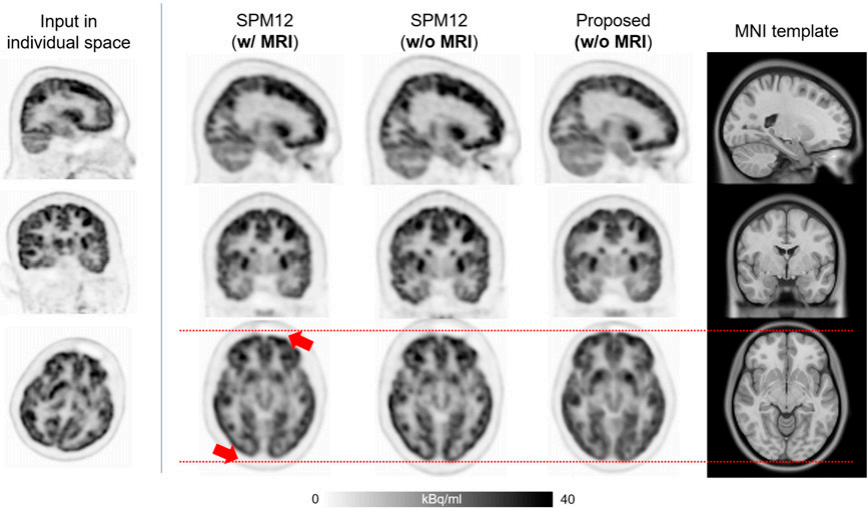
^18^F-FDG PET images of 13-y-old boy with epilepsy, included in internal dataset. Small brain in individual space was not adequately resized by SPM, as indicated by red arrows.

**FIGURE 3.**
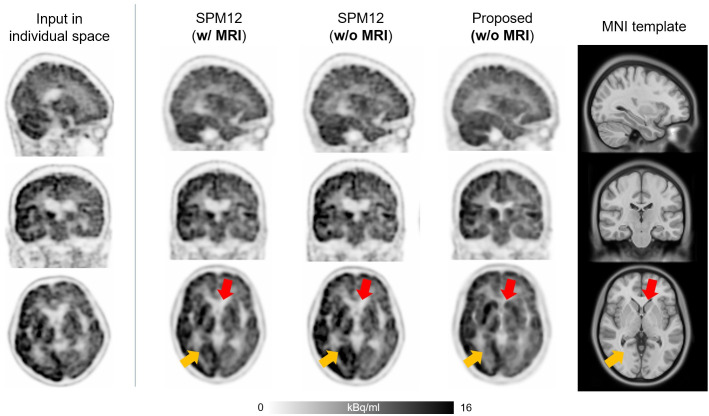
^18^F-FDG PET images of 72-y-old woman from internal dataset, exhibiting striatum atrophy. Although SPM did not perfectly reshape region (red arrows), proposed method showed significant improvement, also outperforming SPM in occipital cortex (indicated by yellow arrows).

**FIGURE 4.**
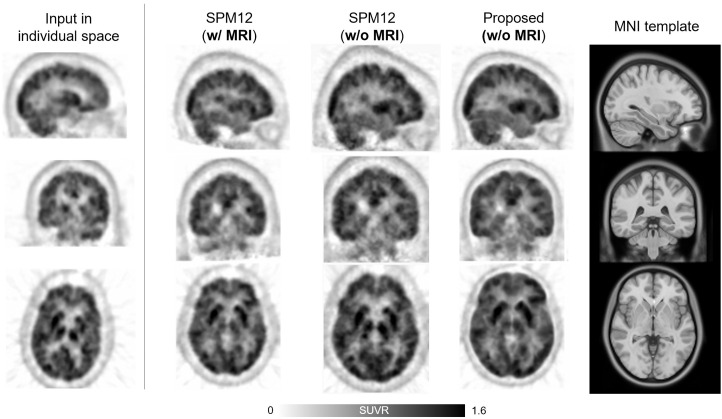
^18^F-FDG PET images of 67-y-old woman selected from an external dataset, exhibiting relatively small brain size, high noise level, and poor spatial resolution.

Comparison between the spatially normalized PET images using the proposed method and SPM is presented in Figures 2–4. Figure 2 shows ^18^F-FDG PET images from the internal dataset of a 13-y-old boy with epilepsy. When SPM was applied, the small brain in individual space was inadequately resized (indicated by red arrows). In contrast, the spatially normalized image using the proposed method exhibited a better match with the 3D MRI template in terms of the brain cortex size.

Figure 3 presents another example from the internal dataset, showing a 72-y-old woman with atrophy in the striatum. Although SPM did not perfectly reshape the striatum (red arrows), the proposed method performed well in deforming it. Furthermore, the proposed method outperformed SPM in the occipital cortex, as indicated by the yellow arrows.

Figure 4 illustrates another case from the external dataset, featuring a 67-y-old woman with a relatively small brain size. The PET image in this case had high noise levels and poor spatial resolution due to reconstruction using the filtered backprojection algorithm. Despite these challenges, the proposed method well compensated for the size difference between the individual brain and the template, outperforming SPM.

**FIGURE 5.**
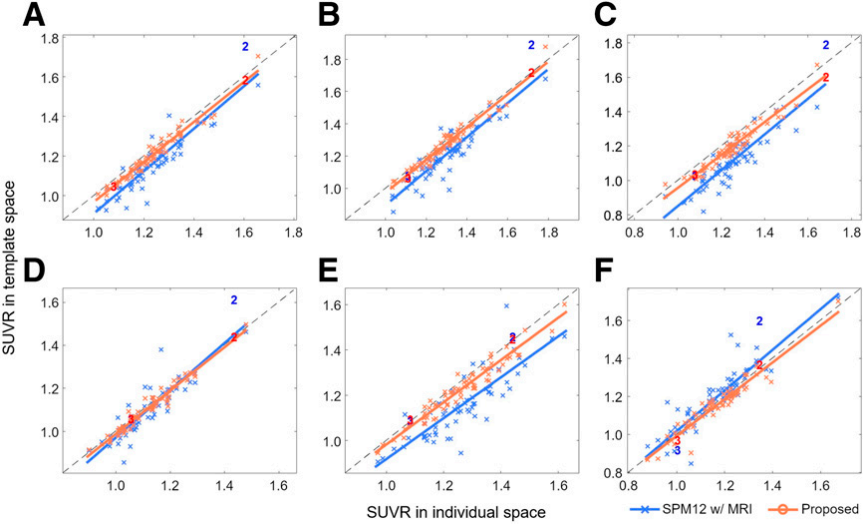
Internal validation. Comparison between MRI-based SUVR quantification in individual space using FreeSurfer (*x*-axis) and spatial normalization–based SUVR quantification in template space using proposed method or SPM with MRI (*y*-axis). Proposed method is represented by orange symbols and regression lines, whereas SPM with MRI is represented by blue symbols and regression lines: global cerebral cortex (A), frontal (B), parietal (C), temporal (D), occipital (E), and subcortex (F).

The improved spatial normalization performance of the proposed method compared with SPM resulted in significantly better SUVR quantification. Figures 5 (internal datasets) and 6 (external datasets) show a comparison between MRI-based SUVR quantification in individual space using FreeSurfer software (*x*-axis, ground truth) and spatial normalization–based SUVR quantification in the template space using the proposed method or SPM with MRI (*y*-axis). The orange and black symbols and regression lines represent the results of the proposed and SPM methods, respectively. The numbers on the plots indicate the corresponding patients illustrated in Figures 2–4. In both internal and external datasets, the proposed method consistently exhibited superior results in terms of variation and correlation across all evaluated brain regions. Notably, SPM’s performance was remarkably poor in several brain regions, such as the parietal and occipital lobes, despite using coregistered MRI for PET spatial normalization. When SPM spatial normalization was performed using only PET images without MRI, SUVR quantification was particularly inaccurate in the subcortex (Supplemental Fig. 1 and 2; supplemental materials are available at http://jnm.snmjournals.org). However, the PET-only framework in SPM appears to be superior to the MRI SPM framework in cortical regions.

**FIGURE 6.**
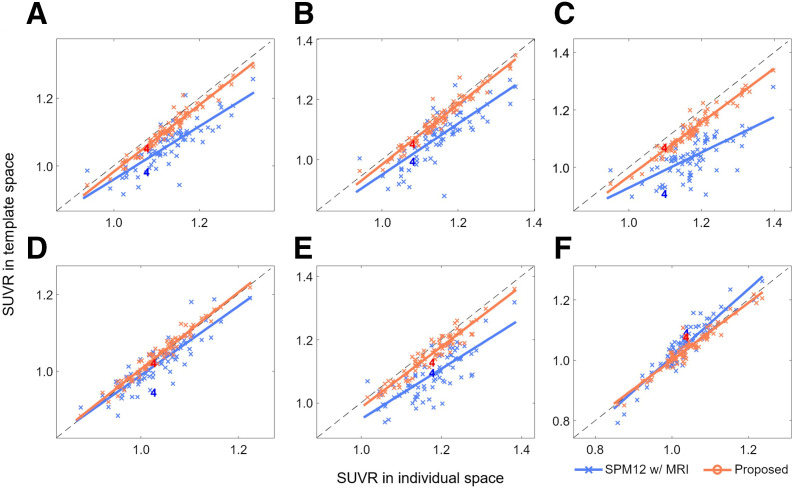
External validation. Comparison between MRI-based SUVR quantification in individual space using FreeSurfer (*x*-axis) and spatial normalization–based SUVR quantification in template space using proposed method or SPM with MRI (*y*-axis). Proposed method is represented by orange symbols and regression lines, whereas SPM with MRI is represented by blue symbols and regression lines: global cerebral cortex (A), frontal (B), parietal (C), temporal (D), occipital (E), and subcortex (F).

**FIGURE 7.**
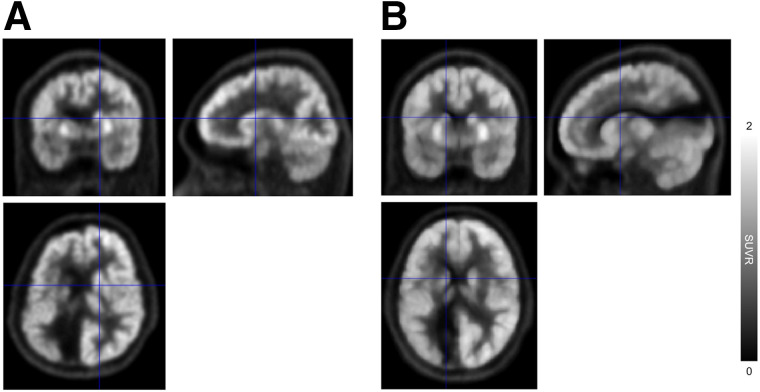
^18^F-FDG PET images of 21-y-old man with hypointense areas that are well preserved in terms of relative size and shape after spatial normalization. Images are shown in individual space before spatial normalization (A) and standard space after spatial normalization (B) using proposed method.

It is also noteworthy that the variation of the proposed method’s SUVR from the regression lines remains consistent regardless of SUVR levels, suggesting its superior performance in hyper- or hypointense areas, such as those affected by severe disease or infarcts. This is illustrated in Figure 7 and Supplemental Figure 3, which show the cases of hypointense areas that are well preserved in terms of relative size and shape after spatial normalization.

The regression parameters and intraclass correlation coefficients (ICCs) are summarized in Table 2 (for internal datasets) and Table 3 (for external datasets). The ICCs between ground truth and SPM with MRI for the internal dataset were 0.77, 0.76, 0.52, 0.88, 0.61, and 0.84 in the cerebral cortex, the frontal, parietal, temporal, and occipital lobes, and the subcortex, respectively, whereas those between ground truth and SPM without MRI were 0.74, 0.69, 0.77, 0.83, 0.75, and 0.49, respectively. In contrast, the proposed method achieved ICCs of 0.96, 0.97, 0.89, 0.97, 0.94, and 0.96 for the same brain regions in the internal dataset. For the external dataset, the performance gap between the 2 methods widens even further. The ICCs were 0.46, 0.49, −0.12, 0.85, 0.29, and 0.89 for SPM with MRI; 0.73, 0.63, 0.69, 0.88, 0.88, and 0.28 for SPM without MRI; and 0.94, 0.95, 0.82, 0.98, 0.91, and 0.96 for the proposed method. The remarkably good correlation and ICC values of the proposed method for the external dataset are noteworthy, considering the dataset’s different ethnic distribution and the use of different PET scanners and image reconstruction algorithms.

**TABLE 2.** Pearson Correlation and ICC Analysis for SUVR of Internal ^18^F-FDG PET Dataset (*n* = 65) Relative to FreeSurfer Approach

	SPM with MRI				SPM without MRI				Proposed			
Region	Slope	*y*-intercept	*R* ^2^	ICC	Slope	*y*-intercept	*R* ^2^	ICC	Slope	*y*-intercept	*R* ^2^	ICC
Cerebral cortex	1.074	−0.166	0.835	0.768	0.872	0.079	0.886	0.743	1.011	−0.043	0.962	0.955
Frontal	1.057	−0.161	0.819	0.764	0.873	0.055	0.895	0.690	1.016	−0.043	0.958	0.968
Parietal	1.040	−0.188	0.782	0.519	0.855	0.109	0.829	0.768	0.957	0.000	0.931	0.885
Temporal	1.097	−0.126	0.816	0.878	0.855	0.112	0.882	0.829	1.006	−0.018	0.959	0.974
Occipital	0.901	0.018	0.716	0.610	0.727	0.277	0.820	0.752	0.932	0.052	0.941	0.936
Subcortex	1.069	−0.052	0.763	0.841	0.896	−0.017	0.855	0.491	0.966	0.027	0.922	0.956

**TABLE 3.** Pearson Correlation and ICC Analysis for SUVR of External ^18^F-FDG PET Dataset (*n* = 78) Relative to FreeSurfer Approach

	SPM with MRI				SPM without MRI				Proposed			
Region	Slope	*y*-intercept	*R* ^2^	ICC	Slope	*y*-intercept	*R* ^2^	ICC	Slope	*y*-intercept	*R* ^2^	ICC
Cerebral cortex	0.784	0.175	0.689	0.455	0.894	0.074	0.872	0.730	0.986	−0.003	0.950	0.938
Frontal	0.849	0.101	0.631	0.486	0.948	−0.008	0.868	0.628	0.997	−0.013	0.934	0.945
Parietal	0.617	0.312	0.425	−0.124	0.837	0.139	0.842	0.690	0.943	0.025	0.927	0.819
Temporal	0.896	0.094	0.766	0.852	0.885	0.101	0.854	0.879	0.996	0.009	0.958	0.975
Occipital	0.795	0.155	0.591	0.292	0.781	0.248	0.802	0.879	0.958	0.028	0.909	0.910
Subcortex	1.123	−0.112	0.859	0.891	1.049	−0.153	0.812	0.282	0.950	0.051	0.914	0.956

## DISCUSSION

In this study, we have developed a highly efficient deep neural network model for fast and accurate automatic spatial normalization of ^18^F-FDG PET images of the brain. This was achieved through transfer learning from a pretrained network model using amyloid PET datasets. By incorporating a well-designed and intensive on-the-fly data augmentation strategy, we could generate the ^18^F-FDG network model using only about 100 datasets, representing just 10% of the amyloid PET datasets used for the original network development (19). Despite the smaller dataset used for ^18^F-FDG network training with parameter fine-tuning, the spatial normalization and SUVR quantification performance were remarkably good, surpassing the widely used conventional SPM method.

Although new and more specific PET radiotracers (e.g., amyloid and tau) have been introduced, ^18^F-FDG PET remains widely used for diverse purposes, such as for evaluating brain tumor malignancy, for localizing epileptic zones, for diagnosing dementia and Parkinson disease differentially, and for staging Alzheimer disease (7–10). Although the visual interpretation of ^18^F-FDG PET images, considering age-dependent physiologic changes in cerebral glucose metabolism, is a standard assessment method, semiquantification of regional glucose metabolism can complement visual assessment. Consequently, various tools for automatic ^18^F-FDG brain PET quantification have been developed, many of which rely on spatial normalization of PET images for fast SUV and SUVR quantification using predefined atlases in the template space (36–39). Conventional approaches involve generating spatially normalized PET images through numeric methods that aim to minimize intensity differences or maximize mutual information between the spatially normalized image and a standard normal template (40). However, these numeric approaches often fail to converge to optimal solutions when dealing with abnormal brain PET images that significantly deviate from the normal template in terms of shape, size, and intensity.

In contrast, the deep neural network–based approach introduced in this study allows for accurate and robust ^18^F-FDG PET spatial normalization, as demonstrated in Figures 2–4. The cases shown in Figures 2 and 4 illustrate how effectively the proposed method handles unusual data with small brain size, whereas the case in Figure 3 highlights the superior performance of the proposed method in transforming small brain regions with atrophy while preserving radiotracer concentration. Notably, the spatial normalization quality of the proposed method was consistent between internal (Figs. 2 and 3) and external (Fig. 4) data, even when using different PET scanners and image reconstruction algorithms. The strong correlation of SUVR quantification (Figs. 5 and 6) between the proposed method and the FreeSurfer-based analysis would be attributed to the high accuracy of the deep neural network–based spatial normalization. It should also be noted that there are potential biases and uncertainties when using MRI-based references, such as errors arising from mismatches between MRI and PET in subject space.

In this study, we used a significantly smaller (10%) number of datasets for the ^18^F-FDG network development compared with the original amyloid network model generation. Furthermore, ^18^F-FDG datasets typically have a wider age distribution than amyloid datasets. However, the ^18^F-FDG SUVR quantification accuracy relative to the ground truth was not notably inferior to that in our previous study with amyloid PET. This highlights the efficacy of transfer learning, which requires a smaller number of datasets than does the original network training, thus increasing the potential for broader use of deep learning–based brain PET spatial normalization techniques for various clinical and research radiotracers. Nevertheless, the development of a more generalized method that requires only a single network model for all PET radiotracers would be an interesting future research topic.

Although our method offers several advantages in terms of accuracy and computational efficiency, it does have some limitations. A drawback is that the normalization process used in our method cannot be reversed. This limitation means that analysis cannot be performed in the patient space with aligned regions, which is often recommended.

## CONCLUSION

In this study, we demonstrate the effectiveness of transfer learning in a deep neural network model for brain PET spatial normalization. Our approach extends a fast and accurate automatic brain PET quantitation method from amyloid radiotracers to ^18^F-FDG. Remarkably, the spatial normalization network model can be generated using just 10% of the datasets compared with the original model generation, making it resource efficient. Therefore, this transfer learning approach will be useful for extending the method to other radiotracers with limited availability.

Our results illustrate that the proposed method yields precise spatially normalized images and provides quantified regional SUVRs for ^18^F-FDG even without matched MRI. Furthermore, in comparison to the widely used conventional method (SPM), the proposed method consistently outperforms on both internal and external test sets. These findings highlight the promising potential of the proposed method for automatic ^18^F-FDG brain PET quantification. To fully establish the clinical utility of the proposed method and explore its applications in diverse patient populations and clinical settings, further investigations will be necessary. Nonetheless, our study paves the way for an innovative and efficient solution in brain PET quantification.

## DISCLOSURE

This work was supported by the Seoul R&BD Program (No. BT200151) through the Seoul Business Agency (SBA) funded by The Seoul Metropolitan Government, the K-Brain Project (No. RS-2023-00264160) of the National Research Foundation (NRF) funded by Ministry of Science and ICT, and the Korea Dementia Research Project (No. HU23C014000) through the Korea Dementia Research Center (KDRC) funded by the Ministry of Health and Welfare and Ministry of Science and ICT. No other potential conflict of interest relevant to this article was reported.

## Notes

KEY POINTS **QUESTION:** Can transfer learning adapt a pretrained deep neural network model from amyloid PET to efficiently spatially normalize ^18^F-FDG PET images without requiring matched 3D T_1_ MRI scans? Does this approach demonstrate resource efficiency and improved performance, making it a viable option for broader application in clinical and research settings? **PERTINENT FINDINGS:** The study successfully uses transfer learning to adapt a pretrained neural network for spatial normalization of ^18^F-FDG PET images, surpassing traditional methods. The proposed approach demonstrates superior performance, indicating its potential for widespread application in clinical and research settings. **IMPLICATIONS FOR PATIENT CARE:** Enhanced spatial normalization performance promises more accurate diagnoses for various brain diseases, translating into improved patient care through timely interventions and neuroimaging advancements.
